# Intermittent Hypoxic–Hyperoxic Training During Inpatient Rehabilitation Improves Exercise Capacity and Functional Outcome in Patients With Long Covid: Results of a Controlled Clinical Pilot Trial

**DOI:** 10.1002/jcsm.13628

**Published:** 2024-11-19

**Authors:** Wolfram Doehner, Azadeh Fischer, Banafsheh Alimi, Jasmin Muhar, Jochen Springer, Christoph Altmann, Per Otto Schueller

**Affiliations:** ^1^ Berlin Institute of Health Center for Regenerative Therapies Charité ‐ Universitätsmedizin Berlin Berlin Germany; ^2^ German Heart Center of the Charite, Department of Cardiology, Campus Virchow, German Centre for Cardiovascular Research (DZHK), partner site Berlin Charité ‐ Universitätsmedizin Berlin Berlin Germany; ^3^ Center for Stroke Research Berlin (CSB) Charité Universitätsmedizin Berlin Berlin Germany; ^4^ Klinik für Kardiologie und Pneumologie Median Klinikum Flechtingen Flechtingen Germany; ^5^ MVZ Cardiologicum Dresden und Pirna Studienzentrum Dresden Dresden Germany

**Keywords:** 6 min walking test, hypoxia, long COVID, rehabilitation, training

## Abstract

**Introduction:**

Long COVID‐19 illness is a severely disabling disease with shortness of breath, weakness and fatigue as leading symptoms, resulting in poor quality of life and substantial delay in return to work.

No specific respiratory therapy has been validated for patients with long COVID. The intermittent hypoxia–hyperoxia training (IHHT) is a respiratory therapeutic modality to improve exercise performance via controlled respiratory conditioning. The purpose of the present study is to investigate the therapeutic effect of IHHT on functional and symptomatic recovery of patients with long COVID syndrome.

**Methods:**

A prospective, controlled, open‐treatment interventional study was conducted in patients with long COVID who were admitted to an inpatient rehabilitation programme. Patients were assigned nonrandomized to receive IHHT in addition to the standardized rehabilitation programme (IHHT group) or standard rehabilitation alone (control group). The IHHT group received supervised sessions of intermittent hypoxic (10–12% O_2_) and hyperoxic (30–35% O_2_) breathing three times per week throughout the rehabilitation period. Primary endpoint was improved walking distance in a 6‐min walk test (6MWT) between study groups. Secondary endpoints were change in stair climbing power, dyspnoea (Borg dyspnoea Scale), fatigue assessment scale (FAS) and change in health‐related quality of life (HRQoL) assessed by patient global assessment (PGA), EQ‐5D analogue scale and the MEDIAN Corona Recovery Score (MCRS). Further assessments included maximum handgrip strength, nine hole peg test, timed up‐and‐go, respiratory function and functional ambulation category (FAC), serum analyses and safety of the intervention.

**Results:**

A total of 145 patients were included in the study (74% female, mean age 53 ± 12 years) and assigned to IHHT (*n* = 70) or standard care (*n* = 75). The 6MWT distance improved 2.8‐fold in the IHHT group compared to the control group (91.7 ± 50.1 m vs. 32.6 ± 54.2 m, ANCOVA *p* < 0.001). Stair climbing power improved 3.7‐fold in the IHHT group compared to controls (−1.91 ± 2.23 s vs. −0.51 ± 1.93 s, *p* < 0.001). Secondary endpoints on dyspnoea, fatigue and HRQoL (PGA, EQ‐5D and MCRS) improved significantly in the IHHT group compared to controls. The IHHT group exhibited a significant decrease in blood pressure, heart rate and increase in haemoglobin levels that was not observed in the control group. No adverse events were observed.

**Conclusion:**

Respiratory treatment with IHHT in addition to a multidisciplinary rehabilitation programme improves functional capacity, symptomatic status and quality of life in patients with disabling long COVID. IHHT has been demonstrated to be safe, well tolerated and feasible to be integrated in an inpatient rehabilitation programme to improve outcome in long COVID.

## 1 Introduction

Long COVID has emerged as a severe complication after SARS‐CoV‐2 infection occurring after both severe and mild courses of the acute infection [1] and is characterized by ongoing symptoms for more than 12 weeks after the acute COVID illness [2]. Prevalence estimates between 10% and 28% have been reported for long COVID [3, 4], but other studies observed long‐term sequelae in up to 50% of patients after COVID‐19 [5, 6, 7]. The most common long COVID symptoms include dyspnoea both at rest and post exertional, cognitive impairments, pain, physical weakness or general fatigue, headaches, sleep disorders and psychological symptoms such as depression or anxiety [8, 9], all accounting for a diminished quality of live (QoL) [10]. Long COVID not only implies prolonged symptomatic illness for the patients but the epidemic proportions of long COVID result in health‐economic and socio‐economic burden due to overwhelmed rehabilitations facilities and long‐term loss of work force. A report from a nationwide German health insurance company showed that loss of work days of patients with long COVID in 2021 was 105 days on average and 168 days in patients with hospitalization for ≥7 days due to COVID‐19 [11]. In comparison, the average loss of workdays of all other patients in 2021 was 15 days. There is a clear and unmet clinical and socio‐economic need to improve recovery and rehabilitation of patients with long COVID.

The most commonly affected organ by COVID‐19 are the lungs with structural and functional pulmonal impairment seen in up to 60% of patients [12, 13]. Pathophysiological changes include alveolar epithelium injury, capillary damage with secondary fibroproliferation and bleeding, septal fibrous proliferation and pulmonary consolidation [14]. It is recommended that patients with continued symptoms and functional impairment of long COVID undergo multidisciplinary rehabilitation [15, 16]. To date, no specific respiratory recovery therapy has been validated for patients with long COVID, and current rehabilitation concepts follow standard nonspecific rehabilitation strategies. Currently, various rehabilitation concepts are being investigated that focus on restoring of respiratory capacity.

Respiratory stimulation by intermittent hypoxia (IH) has emerged as an innovative treatment option, whereby controlled exposure to short‐term intervals of hypoxic breathing results in a conditioning to improve respiratory and cardiovascular function. The mechanisms by which controlled IH exerts stimulation of the respiratory and vascular system are based on the same principles as high altitude training that is known to improve functional capacity in sports athletes [17] as well as in patients with cardiovascular disease [18]. Intermittent hypoxic–hyperoxic therapy (IHHT) in cardiac patients has shown to improve exercise performance [19], cognitive function [20, 21] and QoL [22, 23]. Emerging data suggest that IHHT may be a suitable treatment in rehabilitation and secondary prevention strategies [24].

The impact of IHHT in patients with impaired functional capacity due to long COVID is not known. However, given the pulmonary involvement in COVID‐19 and the demonstrated cardiorespiratory effects of IHHT, a beneficial effect of IHHT in long COVID patients can be hypothesized. The aim of the present study was to investigate the effect of IHHT as a complementary treatment during multidisciplinary rehabilitation to improve functional capacity and outcome in patients with long COVID.

## 2 Methods

### 2.1 Study Design and Patients

This pilot study was a prospective, controlled, open‐treatment, single‐centre, interventional study in hospitalized patients at the rehabilitation hospital MEDIAN Klinikum Flechtingen, Germany. Consecutive patients admitted to the rehabilitation centre between July 2021 and September 2022 with a primary diagnosis of debilitating long COVID syndrome were included in the study. Patients enrolled in the study were older than 18 years, were diagnosed with long COVID Syndrome (ICD‐10), had clinical symptoms of impaired functional capacity (fatigue, dyspnea on exertion and inability to work) and signs of respiratory insufficiency (Borg dyspnoea scale ≥3 or SpO_2_ ≤ 85%) and were capable to perform a 6‐min walking test (6MWT) and to tolerate a IHHT treatment using a breathing mask during the respiratory treatment sessions. Patients with acute infective illness, uncontrolled cardiovascular disease, ongoing immune suppression therapy or those judged by the physician unable to adhere to the study procedures were excluded. Patients were assigned as clinically judged by the attending physician to the IHHT or control group. The protocol was approved by local Ethics Committee, and written informed consent was obtained from patients included in this study.

### 2.2 Intermittent Hypoxic Breathing Intervention

Patients hospitalized for a rehabilitation programme were assigned equally to receive IHHT on top of the standard multidisciplinary rehabilitation programme (IHHT group) or the rehabilitation programme alone (control group). Patients in the IHHT group received three IHHT sessions per week on alternate days throughout the inpatient rehabilitation period (~5 weeks) using the breathing therapy device ReOxy (Aimediq S.A., Luxembourg). Before the start of treatment, each patient underwent testing for cardiorespiratory response to hypoxia to determine individualized trigger points for the automated safety configuration of the device during the following training sessions (blood oxygen saturation, SpO_2_, assessed by continued finger pulse oximeter, heart rate, air flow and blood pressure). IHHT sessions (45 min) included six to eight cycles of repeated short‐term (3–5 min) episodes of hypoxic air breathing (10–12% O_2_ breathing air), followed by hyperoxic air breathing (30–35% O_2_) [19]. The advanced biofeedback technology of the ReOxy device enables real‐time adjustment of the hypoxic load, based on continuous monitoring of individual SpO_2_ levels, heart rate, and the breathing volume. This adaptive approach accounts for precise and individualized modulation of hypoxic and hyperoxic exposure according to the patient's physiological state. IHHT was administered on top of the standardized multidisciplinary rehabilitation programme that included modules of physical rehabilitation (breathing exercise, endurance and strength training), relaxation techniques, education, occupational therapy, psychological counselling and optimized management of comorbidities (see the Supporting Information).

### 2.3 Clinical and Functional Assessments

Changes in functional capacity and patients subjective and objective assessment of symptoms and health were assessed in all patients at baseline and at the end of the rehabilitation programme with a battery of functional tests, scores and questionnaires. Functional capacity was assessed by 6MWT, stair climbing power test, timed up‐and‐go test, maximum handgrip strength test (Kern MAP 80 K1, Kern und Sohn GmbH, Balingen, Germany) and nine‐hole‐peg test for assessment of upper extremity motor function (dexterity). Mobility as assessed by global functional ambulatory category (FAC). Respiratory function was assessed by spirometric testing (PowerCube‐Body, Ganshorn GmbH, Niederlauer, Germany).

Patients physical and mental capacity on admission and discharge was assessed by the fatigue assessment scale (FAS), and symptomatic dyspnoea was assessed by the modified Borg scale. Patients subjective global well‐being was assessed by patient global assessment (PGA); physical, psychic and social functions were assessed by the MEDIAN Corona Recovery Score (MCRS) [25]; and health‐related quality of life (HRQoL) was assessed using the European Quality of Life 5 dimensions questionnaire and analogue scale (EQ‐5D).

Assessment of further clinical variables included change in cardiovascular functions (blood pressure, heart rate and heart rate variability) and haematologic and biochemical variables. Biochemical parameters were assessed in the standard clinical laboratory. For safety assessment, adverse events in all patients and tolerability of the IHHT were recorded. All functional assessments and questionnaires were routine methods of clinical assessment during the rehabilitation programme and were performed according to predefined standard operations protocols by experienced technicians of the clinical centre who were unaware of the group assignment of the patients.

### 2.4 Primary and Secondary Endpoints

The primary endpoint was improvement in walking distance in a 6 min walking test at the end of the inpatient rehabilitation period compared between treatment groups. Secondary endpoints included change in stair climbing power, change in symptomatic status assessed modified Borg Scale for symptomatic dyspnoea, by FAS, PGA, MCRS and change in HFQoL by EQ‐5D visual analogue scale. Further study related analyses included changes in functional tests (maximum handgrip, nine‐hole peg test, timed up‐and‐go, functional ambulatory capacity and FAC) assessments of respiratory capacity (FEV1, PEF and VC) and changes in clinical and biochemical variables.

### 2.5 Statistical Analysis

Values are expressed as medians and interquartile ranges (IQRs), or counts and percentages, as appropriate. Group comparisons of continuous variables were performed using the Student's *t*‐test for continued variables and Pearson's Chi‐squared test for categorical data as appropriate. Paired *t*‐test was used for comparison of paired variables, assessing changes of functional capacity or clinical variable within groups from baseline to end of rehabilitation.

The outcome analysis was based on intention to treat analysis of all included patients who were assigned to either of the two treatment arms. The primary endpoint (change in walking distance in 6MWT at the end of the inpatient rehabilitation comparing the IHHT group vs. control group) was analysed by covariance adjusted for baseline (ANCOVA). The treatment effect on functional outcomes was further assessed as change from baseline comparing both the treatment groups. Sample size calculation was based on group differences for the primary endpoint. A sample size of 64 patients per treatment group was calculated to achieve 80% power with a two‐sided equal‐variance *t*‐test with α = 0.05 to detect a minimal difference between treatment groups of 40 m change in walking distance. A distance of ≥35 m is regarded as minimum of a clinical meaningful improvement [26]. Allowing for a drop out of 7%, a total of 70 patients was planned to be enrolled in each group. All statistical tests were two‐tailed, and a two‐sided *p* value of 0.05 was considered for significance. The statistical analyses were performed using Statistical Package for the Social Sciences (SPSS) version 22.0 (SPSS Inc., Chicago, Illinois, United States).

## 3 Results

### 3.1 Clinical Characteristics

A total of 145 patient consecutively admitted to the inpatient rehabilitation with long COVID syndrome were enrolled in the study (mean age 53.2 ± 11.6 years, female: 74%). Of these, 70 patients were assigned to the IHHT arm, and 75 patients were assigned to the control arm receiving the standard rehabilitation programme. Mean rehabilitation duration was 27 ± 6 days. The clinical characteristics of the study population of both treatment groups are shown in Table 1. Both treatment groups did not differ significantly in main clinical variables (sex distribution, body composition, blood pressure, respiratory function and medical treatments); however, differences between treatment groups were observed for age, length of hospitalization and a higher prevalence of coronary artery disease and COPD in the control group.

**TABLE 1:** Baseline characteristics of patients per treatment group (mean ± SD).

Characteristics	IHHT group (*n* = 70)	Control group (*n* = 75)	*p* value
Age, years	50.8 ± 10.6	55.1 ± 11.7	0.021
Gender, male (%)	18 (25.7)	19 (25.3)	ns
Weight, kg	78.5 ± 18.7	82.1 ± 21.6	ns
BMI, kg/m^2^	27.3 ± 5.4	28.4 ± 6.9	ns
Systolic BP, mmHg	129 ± 14	128 ± 18	ns
Diastolic BP, mmHg	82 ± 8	81 ± 9	ns
HR, bpm	78 ± 9	76 ± 12	ns
FEV1%	83 ± 15	85 ± 21.1	ns
PEF, L/s	76 ± 16	79 ± 23	ns
Vital capacity, L	85 ± 14	90 ± 20	ns
Length of stay in rehab, days	29.5 ± 6.3	24.9 ± 5.5	<0.001

Comorbidities			
Coronary artery disease	5 (7.1)	15 (20.0)	0.02
Heart failure	1 (1.4)	3 (4.0)	ns
Arterial hypertension	21 (30.0)	31 (41.3)	ns
Atrial fibrillation	4 (5.7)	1 (1.3)	ns
Diabetes mellitus	4 (5.7)	6 (88.0)	ns
COPD	2 (2.9)	10 (13.3)	0.02
Current smoker	5 (7.1)	7 (9.3)	ns
Hypothyroidism	7 (10.0)	5 (6.7)	ns
Depression	3 (4.3)	1 (1.3)	ns

Medications, *n* (%)			
ß blocker	12 (17.1)	12 (16.0)	ns
ACE inhibitors	15 (21.4)	19 (25.3)	ns
Calcium channel blockers	5 (7.1)	13 (17.3)	ns
Antiplatelet therapy	5 (7.1)	6 (8.0)	ns
Diuretics	2 (2.8)	5 (6.6)	ns
Thyroid hormone replacement	6 (8.5)	5 (6.6)	ns

Biochemical variables			
Hb, mmol/L	8.6 ± 0.7	8.6 ± 0.8	ns
CRP, nmol/L	30.4 ± 31.1	45.2 ± 82.1	ns
FBS, mmol/L	5.4 ± 1.2	5.8 ± 5.0	ns
Insulin, μE/mL	12.4 ± 6.5	12.7 ± 10.8	ns
Cholesterol, mmol/L	5.8 ± 0.8	6.3 ± 4.2	ns
LDL, mmol/L	3.7 ± 0.8	3.9 ± 2.6	ns
HDL, mmol/L	1.8 ± 0.5	1.6 ± 0.5	<0.01
Creatinine, μmol/L	72.2 ± 10.1	72.7 ± 13.1	ns
GFR, mL/min 1.73 m^2^	95.9 ± 11.3	87.6 ± 15.1	<0.001

Abbreviations: COPD, chronic obstructive pulmonary disease; CrP, C‐reactive protein; FBS, fasting blood sugar; GFR, glomerular filtration rate; Hb, haemoglobin; HDL, high density protein; HR, heart rate; LDL, low‐density protein.

### 3.2 Primary and Secondary Outcomes

**FIGURE 1:**
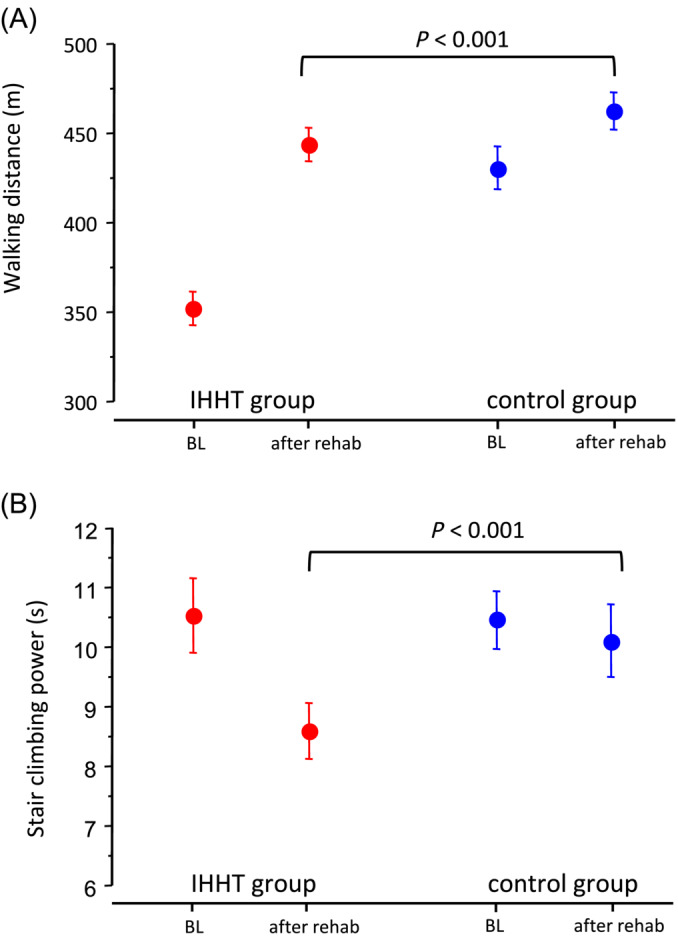
Treatment effect of IHHT on functional capacity in patients with long COVID. (A) walking distance in 6 min walking test (primary endpoint) and (B) stair climbing power (secondary endpoint).

The primary endpoint is shown in Figure 1 and Table 2. Both treatment groups showed significant improvement in the walking distance at the 6MWT after the rehabilitation compared to baseline. The improvement in walking capacity was 2.8‐fold greater in the IHHT group compared to standard care (91.7 ± 50.1 m vs. 32.6 ± 54.2 m, ANCOVA, adjusted for baseline *p* < 0.001).

**TABLE 2:** Comparison of functional capacity in the IHHT treatment group versus control group.

MCRS Variables	IHHT group		*P* value	Control group		*p* value	*P* value IHHT vs. control*
	Baseline	After rehab		Baseline	After rehab		
**Functional tests**							
6 MWT, m	352 ± 75	443 ± 77	*P* < 0.001	430 ± 81	462 ± 89	*P* < 0.001	*P* < 0.001
Stair climbing power, s	10.5 ± 5.3	8.5 ± 4.0	*P* < 0.001	10.5 ± 3.9	10.0 ± 4.7	0.04	*P* < 0.001
Max handgrip R, Kg	26.2 ± 8.2	27.4 ± 7.8	0.01	22.5 ± 9.7	23.2 ± 10.5	ns	
Max handgrip L, Kg	24.9 ± 8.1	26.3 ± 7.3	0.001	21 ± 9.2	21.4 ± 9.5	ns	
Nine‐hole peg R, s	21.7 ± 3.1	20.4 ± 2.7	*P* < 0.001	21.8 ± 4.1	22 ± 4.5	ns	
Nine‐hole peg L, s	21.8 ± 3.4	20.9 ± 3.3	0.002	23.1 ± 4.4	22.7 ± 4.2	ns	
Timed up‐and‐go, s	7.9 ± 2.4	6.9 ± 2.1	*P* < 0.001	8.2 ± 2.7	7.6 ± 2.8	*P* < 0.001	ns
FAC score	4.97 ± 0.17	4.98 ± 0.12	ns	5.0 ± 0.0	5.0 ± 0.0	ns	
FEV1, %	82.9 ± 15.2	85.2 ± 13.2	*p* = 0.009	85.1 ± 20.9	86.7 ± 18.9	ns	
PEF, %	76.3 ± 15.9	79.3 ± 13.8	*p* = 0.005	77.7 ± 23.5	78.3 ± 21.4	ns	
VC, %	85.2 ± 14.2	87.5 ± 12.6	*p* = 0.009	89.6 ± 20.6	89.4 ± 18.7	ns	
**Symptomatic scores and subjective health**							
Borg Scale	3.6 ± 1.9	1.9 ± 1.4	*P* < 0.001	3.1 ± 2.1	2.2 ± 1.8	*P* < 0.001	*P* < 0.01
FAS	39.3 ± 4.4	30.4 ± 5.8	*P* < 0.001	34.2 ± 5.2	37.8 ± 5.0	P < 0.001	*P* < 0.001
PGA		6.6 ± 0.6			5.7 ± 0.8		*P* < 0.001
EQUATION 5D analogue scale	38.3 ± 8.3	69.7 ± 11.6	*P* < 0.001	86.7 ± 7.0	89.7 ± 5.9	*P* < 0.001	*P* < 0.001
MCRS global score	22.4 ± 5.8	12.1 ± 6.2	*p* < 0.001	17.1 ± 9.2	15.0 ± 9.2	*p* = 0.001	*P* < 0.001
MCRS Somatic score	14.7 ± 2.2	6.3 ± 2.6	*p* < 0.001	5.3 ± 3.9	4.2 ± 3.1	*p* < 0.001	
MCRS Psychological score	6.6 ± 4.3	4.3 ± 3.6	*p* < 0.001	7.1 ± 6.0	5.8 ± 5.6	*p* < 0.001	
MCRS Life Events score	1.2 ± 2.0	1.3 ± 2.2	ns	4.3 ± 3.3	4.4 ± 3.7	ns	

Abbreviations: EQUATION 5D, European Quality of Life 5 dimensions questionnaire; FAC, functional ambulatory category; FAS, fatigue assessment scale; FEV1, forced expiratory volume 1st second; MCRS, the MEDIAN Corona Recovery Score; PEF, peak expiratory flow; PGA, patient global assessment; VC, vital capacity.

* ANCOVA, adjusted for baseline.

**FIGURE 2:**
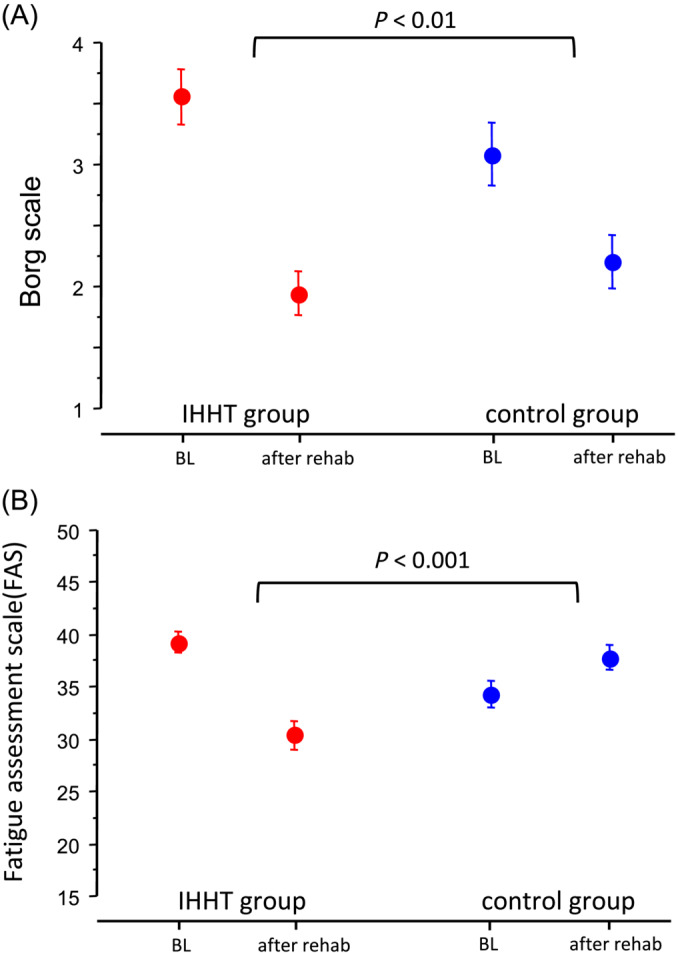
Treatment effect of IHHT symptomatic status in patients with long COVID. (A) Borg Scale of dyspnoea and (B) Fatigue assessment (FAS, secondary endpoints).

**FIGURE 3:**
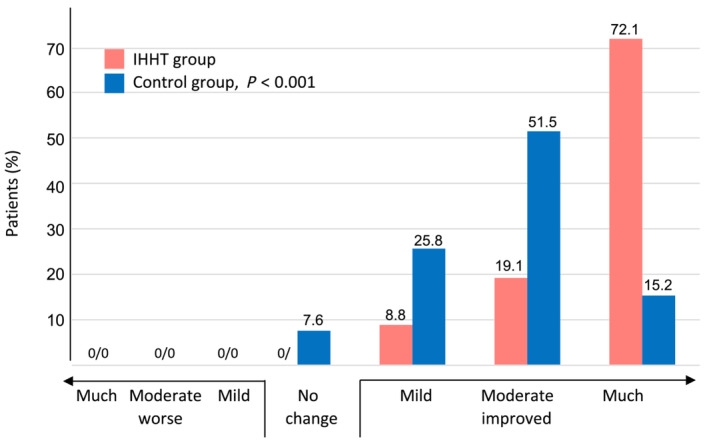
Treatment effect of IHHT on patient global assessment (PGA) in patients with long COVID at discharge from inpatient rehabilitation (secondary endpoint).

The secondary endpoint stair climbing power improved significantly after the rehabilitation programme in both treatment groups, the IHHT group showed a significant benefit with a 3.7‐fold higher improvement compared to controls (IHHT group, −1.91 ± 2.23 s vs. controls −0.51 ± 1.93 s; *p* < 0.001, Figure 1). Further secondary endpoints: The Borg scale of symptomatic dyspnoea improved significantly in both treatment groups. The improvement from baseline was twofold greater in the IHHT group compared to controls (Score −1.7 ± 1.2 vs. −0.8 ± 1.4, *p* < 0.001, Figure 2). FAS improved in the IHHT group, but a deterioration was observed in the control group (FAS ‐8.9 ± 5.2 vs. +3.6 ± 3.2, *p* < 0.001, Figure 2). PGA showed a significant benefit of the IHHT treatment compared to the control group (mean PGA 6.6 ± 0.7 vs. 5.7 ± 0.8, *p* < 0.001, Figure 3).

**FIGURE 4:**
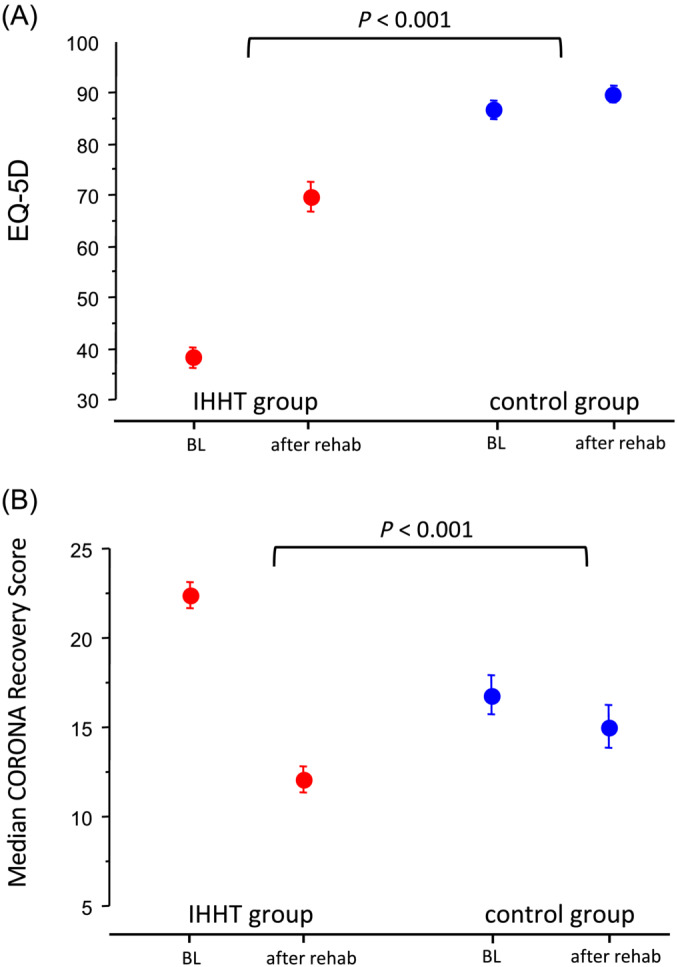
Treatment effect of IHHT on health‐related quality of life in patients with long COVID. (A) EQ‐5D analogue scale and (B) Median CORONA Recovery Score (MCRS, secondary endpoints).

HRQoL showed a significant greater improvement in the EQ‐5D analogue scale in the IHHT group compared to the control group (31.3 ± 10.6 vs. 2.9 ± 2.8, *p* < 0.001, Figure 4). MCRS improved after the rehabilitation in both treatment groups in the somatic category and in the psychological category but not in the live event category. The improvement of the individual categories and of the total MCRS (−10.3 ± 3.7 vs − 2.1 ± 4.6; *p* < 0.001) was significantly greater in the IHHT group compared to the control group (Table 2 and Figure 4).

Further changes in functional assessments are shown in Table 2: Max handgrip strength and nine‐hole peg tests of both hands improved significantly in the IHHT group, but no such improvement was observed in the control group after the rehabilitation. The timed up‐and‐go test improved in both treatment groups to a similar degree without showing a significant benefit in the IHHT group. The FAC did not show a significant change in either of the treatment groups after the rehabilitation and no significant difference between treatment groups. Respiratory function as assessed by spirometric testing improved in the IHHT group after the rehabilitation, but no improvement was observed in the control group.

### 3.3 Clinical Measurements and Biochemical Variables

Clinical measurements and biochemical variables before and after the rehabilitation period are shown in Table 3. The IHHT group showed significant improvement of blood pressure, resting heart rate, resting SpO_2_ (all *p* < 0.001) and in body weight (*p* < 0.01), but no changes in these parameters were observed in the control group.

**TABLE 3:** Cardiorespiratory measurements and biochemical variables before and after rehabilitation in study groups.

Variables	IHHT group		*P* value	Control group		*p* value
	Baseline	After rehab		Baseline	After rehab	
**Clinical measurements**						
Systolic BP, mmHg	129.6 ± 14.2	123.6 ± 10.6	*p* < 0.001	127.7 ± 17.6	125.1 ± 12.3	ns
Diastolic BP, mmHg	82.5 ± 7.9	78.3 ± 5.3	*p* < 0.001	80.9 ± 9.4	79.7 ± 7.5	ns
HR, bpm	78.4 ± 8.7	75.6 ± 8.1	*p* < 0.001	76.3 ± 11.7	77.6 ± 8.5	ns
O_2_ saturation, %	97.1 ± 1.4	98 ± 1	*p* < 0.001	97.9 ± 1.1	98 ± 0.8	ns
Weight, kg	78.5 ± 18.7	77.3 ± 17.7	*p* = 0.01	82.4 ± 21.6	82.4 ± 21.2	ns
**Biochemical variables**						
Hb, mmol/L	8.5 ± 0.7	8.7 ± 0.6	*p* < 0.001	8.5 ± 0.7	8.6 ± 0.7	ns
CRP, nmol/L	30.4 ± 31	19.5 ± 17.3	*p* < 0.001	46.3 ± 83	42.7 ± 128.2	ns
FBS, mmol/L	5.4 ± 1.1	5.1 ± 0.6	*p* = 0.001	5.8 ± 5	5.1 ± 0.9	ns
Insulin, μE/mL	12.4 ± 6.4	12.2 ± 4.5	NS	12.5 ± 10.7	12.7 ± 11.1	ns
Cholesterol, mmol/L	5.8 ± 0.7	5.4 ± 0.7	*p* < 0.001	6.3 ± 4.3	5.8 ± 4.4	*p* < 0.001
LDL, mmol/L	3.6 ± 0.8	3.2 ± 0.7	*p* < 0.001	3.6 ± 1	3.3 ± 0.9	*p* = 0.002
HDL, mmol/L	1.8 ± 0.4	1.8 ± 0.3	NS	1.5 ± 0.4	1.4 ± 0.4	*p* = 0.02
Creatinine, μmol/L	72.2 ± 10.1	70.8 ± 7.3	NS	72.3 ± 12.6	72.3 ± 14.3	ns
GFR, mL/min 1.73 m^2^	95.9 ± 11.3	97.1 ± 8	NS	87.9 ± 14.7	87.5 ± 15.1	ns

A significant increase of Hb and decrease of fasting glucose levels and CRP after the rehabilitation were observed in the IHHT group (all *p* ≤ 0.001) but not in the control group. Lipid profiles improved in both treatment groups, and kidney function was not changed.

### 3.4 Safety and Tolerability of IHHT

The IHHT treatment was well tolerated in all subjects. No adverse events or serious adverse events were recorded in either of the treatment groups. No dropout of patients was recorded due to intolerance to the respiratory treatment using a breathing mask.

## 4 Discussion

The main finding from this study is that intermittent hypoxic–hyperoxic training during in‐patient rehabilitation improved functional capacity, symptomatic status and HRQoL of patients with long COVID syndrome. In this controlled study, the primary endpoint of walking distance in a 6 min walking test improved by 92 m (mean) in the IHHT group compared to 33 m in the control group. Improved functional capacity was further demonstrated in a number of functional tests and symptomatic scores in patients treated with IHHT compared to control patients who received the standard rehabilitation programme. Such functional improvements included stair climbing power, maximum handgrip strength, nine‐hole peg test and respiratory functions (FEV1, PEF and VC). This is the first report to demonstrate in a clinical study that IHHT improved functional capacity in patients with long COVID.

Significant symptomatic improvement and improved HRQoL after IHHT were demonstrated with a range of functional scores for dyspnoea (Borg dyspnoea scale), FAS, PGA and EQ‐5D analogue scale. The MCRS as an integrative score of somatic and psychological symptomatic status and disease‐related social implications showed a significant improvement of patients after IHHT compared to standard rehabilitation.

The observed beneficial effects on functional capacity are in line with previous reports in patients with cardiovascular disease [19], in geriatric patients [20] and in sport athletes [17]. The study extends this observation to patients with long COVID syndrome and underscores the potential impact of the IHHT as treatment strategy in long COVID rehabilitation. A major goal of the inpatient rehabilitation is to restore working capacity and accelerate return to work for the patient after the COVID illness. Data from health insurance registries show that the loss of working days due to COVID related long‐term disability was 105 days on average as compared to 15 days on average for all other patients [11]. It can be reasonably assumed that improved outcome after a multidisciplinary rehabilitation that includes IHHT will not only result in an enhanced functional capacity and HRQoL for the patients but may as well lead to an improvement in their social participation, and a faster return to work. The socioeconomic aspect of improved rehabilitation outcome warrants further investigation.

Clinical and biochemical variables improved in the IHHT group, but this effect was not observed in the control group. Blood pressure (systolic and diastolic BP), heart rate, O_2_ saturation at rest and body weight improved significantly in the IHHT group. Haemoglobin concentration increased, and plasma levels of CRP and fasting blood sugar decreased significantly in the IHHT group. These changes are in line with previous reports on improved blood pressure [27] and metabolic measurements [28]. The change of these variables did not reach the level of significance in the control group; however, similar trends were observed. Further studies are warranted to reveal if these effects of IHHT may yield clinically meaningful benefits for these patients.

### 4.1 Method Discussion

The effect on functional capacity was assessed by a battery of tests and scores that ranged from global functional tests (FAC) and health‐related measures (PGA) to highly selective functional testing (handgrip strength and nine‐hole peg test). The primary endpoint of change in walking distance in 6MWT represents a robust and validated test and clinically highly meaningful measure in patients with impaired functional capacity. 6MWT is applied as primary or secondary endpoint in multiple studies [29, 30]. An improved walking difference of ≥35 m is considered the threshold to demonstrate clinically meaningful improved walking capacity in patients with cardio‐pulmonary diseases such as heart failure or pulmonary hypertension [26, 31]. Notably, an improved walking distance of 32.6 m was observed in the control group of the current study, which confirms the beneficial effect of the applied standardized rehabilitation programme. An improved walking distance of 91.7 m was observed, however, in the IHHT group, indicating an 2.8‐fold stronger improvement compared to the standard rehabilitation programme. The IHHT group showed further improvement on a range of tests (stair climbing power, handgrip strength, nonhole peg test and respiratory function) but not in all functional tests. The timed up‐and‐go test showed improvement in both treatment groups but did not demonstrate a difference between groups, and the FAC did not show improvement of function in either group. The FAC measurement showed results at or near the maximum achievable value of this test (i.e., 5 points). Therefore, a ceiling effect maybe concluded for the FAC that renders the test unsuitable to quantify functional improvement in these patients. As this study was intended as pilot to inform subsequent randomized studies, it may be concluded that 6MTW is a highly applicable test to study treatment effects in this population. In turn, the FAC and timed up and go tests were found unsuitable to assess treatment benefits in this study setting.

### 4.2 Mechanistic Discussion

Evidence has accumulated in recent years to demonstrate IHHT as a safe and efficient nonpharmacological intervention to improve physical and cognitive performance in health subjects as in patients with cardiac disease and impaired functional capacity [32]. The treatment concept applies a similar treatment principle as high altitude training for sports athletes to improve functional capacity, and the interventional approach ‘live low‐train high’ has emerged as an effective training modality [17, 33, 34]. The utilization of IHHT seems of particularly value in combination with physical rehabilitations programme in cardiovascular patients where the continued supervised treatment over several weeks under controlled clinical conditions may be ensured such as applied here during an inpatients rehabilitation programme [24].

The intermittent hypoxic conditioning has been demonstrated to account for a range of adaptive processes of the cardiovascular system as well as the cellular oxidative metabolism. The physiologic principle of IHHT is understood to be mediated via activation of the hypoxia inducible factor‐1 alpha (HIF‐1α) [35], which is indeed increased following IHHT [36]. HIF‐1α has a central role in the regulation of cellular respiration, resulting in improved mitochondrial efficacy to utilize oxygen for energy production [37]. HIF‐1α is a master transcription factor that regulates over 100 genes involved in cellular adaptive response to hypoxia. Multiple pathways are activated in adaptation to controlled hypoxic stimulation that all contribute to improved oxidative capacity such as upregulated erythropoiesis, angiogenesis, antioxidant enzymes, control of inflammatory activation, accelerated tissue migration of immune cell, mitochondrial biogenesis and energy substrate metabolism [38]. HIF‐1α mediated pathways further influence endothelium function to control vascular tone, neuro‐regenerative effects, cell growth and differentiation, cell survival and apoptosis. More detailed pathophysiologic studies are warranted to investigate if IHHT may as well alleviate pulmonal and myocardial injury in patients after COVID illness [39].

Notably, pathophysiologic studies suggest that the oxygen gradient between alternate O_2_ levels may have a stronger stimulating effect on HIF‐1α activation than the low oxygen level alone [40, 41, 42]. This would support the notion that the hypoxia–hyperoxia intermittent stimulation seems to yield a stronger effect than hypoxic episodes alone [32, 43]. The dose of hypoxia as applied in the current treatment protocol [44], the continued monitoring of patients with automated switch from hypoxic to hyperoxic breathing in repeated cycles and the dosing of training on alternate days throughout the hospitalized rehabilitation period have shown to be effective, safe and well tolerated by the patients. In fact, a positive subjective feedback of strengthened and energetic feeling after treatment sessions was reported by patients in the IHHT group.

### 4.3 Limitations

Our pilot study was planned as an open‐treatment, parallel‐group interventional comparison, and the limitations of the unblinded design clearly need to be acknowledged. Patients were assigned to the study groups according to clinical judgement (nonrandomized) and differences at baseline between study groups observed in some of clinical characteristics and biochemical variables may be explained by this. Patients in the IHHT group were on average 5 years younger, had a lower prevalence of comorbidities such as coronary artery disease or COPD, higher HDL levels and better renal function. In turn, functional testing and symptomatic scores showed more severe symptomatic status in the IHHT group compared to controls at baseline in some but not all tests. In our analysis, the inequality between groups at baseline was addressed by adjusting the assessment of treatment effect on the functional endpoints and other variables for the baseline values. Notably, the constant finding of a beneficial effect of IHHT treatment across a range of functional tests and symptom scores supports the robustness of the findings and renders it unlikely that the findings are a mere matter of chance. It cannot be fully excluded, however, that the baseline differences and the open‐treatment design may affect the observed treatment effect in our study. Our study is therefore considered a pilot study with the intention to provide real life clinical treatment data to support the hypothesis and in preparation of a controlled, blinded, randomized clinical trial. The robust and consistent findings on the benefit of IHHT in patients with long COVID syndrome warrant further investigation and validation in a randomized and blinded setting. Potential combined effects of IHHT with novel metabolic treatment options such as SGLT2‐inhiotors on health status warrant further investigation [45].

## 5 Conclusion

Intermittent hypoxic–hyperoxic training in patients with long COVID and severely impaired functional capacity has been shown to improve functional capacity, health‐related quality of life and objective and subjective symptomatic status. IHHT has been demonstrated to be safe, well tolerated and feasible to be integrated in a hospitalized interdisciplinary rehabilitation programme to improve outcome after long COVID. Beneficial effects on measures of cardiovascular function and metabolism are promising. The therapeutic benefit of IHHT in patients with long COVID warrants further validation in a controlled clinical study.

## Conflicts of Interest

W. Doehner reports consulting fees and speaker honoraria from Bayer, Boehringer Ingelheim, Boston Scientific, Cardiomatics, Aimediq, Medtronic, Vifor Pharma, travel support from Pharmacosmos and research support to the Institute from EU (Horizon2020), German Ministry of Education and Research, German Center for Cardiovascular Research, German Pension Insurance (regional Mitteldeutschland) Boehringer Ingelheim, Vifor Pharma. C. Altmann reports consulting fees, speaker honoraria and research support from Pfizer, Amgen, Amarin, Novartis. P. Schüller declares consulting fees, speaker honoraria and research support from Pfizer, Amgen, Amarin and Novartis. A. Shafieesabet, B. Alimi, J. Muhar and J. Springer report no conflict of interest.

## Supporting information


**Data S1.** Supporting Information.
