# Supplementary material for: Intermittent Hypoxic–Hyperoxic Training During Inpatient Rehabilitation Improves Exercise Capacity and Functional Outcome in Patients With Long Covid: Results of a Controlled Clinical Pilot Trial
Source: J Cachexia Sarcopenia Muscle. 2024 Nov 19;15(6):2781–91. doi: 10.1002/jcsm.13628 (PMC11634465; doi:10.1002/jcsm.13628)
Supplement: Supplementary file 1 — Data S1. Supporting Information. [file JCSM-15-2781-s001.docx]

**Standardized multidisciplinary rehabilitation program for patients with Long COVID**

1. Diagnostic testing

Diagnostic testing involved structured assessments using the Patient Health Questionnaire—9

(PHQ-9) and the Generalized Anxiety Disorder Questionnaire– 7 (GAD-7) that allow to detect

symptoms of depression and anxiety (Hinz et al., 2017; Levis et al., 2019). Measurement of

routine laboratory parameters, electrocardiography, echocardiography, body

plethysmography, and the 6MWD.

2. Physical rehabilitation

Physical rehabilitation was comprised of different training modalities. (1) Breathing exercises

included inspiratory and expiratory muscle training, resonance frequency breathing,

diaphragmatic breathing, and pursed-lip breathing. (2) Endurance and strength training was

performed under the control of arterial oxygen saturation (SPO2), heart rate (HR), and blood

pressure (HR). The training consisted of gradual increased aerobic exercises, avoidance of

overexertion, balance and coordination training, individual working training, motor assisted

movement training, and cycle-based endurance training. (3) Physical therapy included

electrotherapy, fango packs, hydrotherapy, and inhalations. (4) Occupational therapy consisted

of cognitive training, functional training, activities of daily life (ADL) training, independence in

selfcare, and fine motor skills.

3. Interdisciplinary educational program

The interdisciplinary educational program of the participants included information about

COVID-19 and Long COVID, coping and pacing strategies, self-management, and acceptance of

the disease. It further included the learning of setting achievable targets and adequate rest

periods and breaks in activities, as well as avoiding overexertion, improving nutrition, acquiring

a healthy lifestyle, and handling health emergencies. Also, medication management,

management of comorbidities, and general hygiene were included in the interdisciplinary

educational program.

4. Psychosocial support

The psychosocial support included patient consultation with a psychologist and the work of a

social worker; Comprising individual counselling to cope with the disease, group therapy for

disease processing, a peer support group for Long COVID patients, techniques for stress control

and relaxation on the one hand, and organization of aftercare, information about support

options, home health care, social reintegration into family, daily life and work on the other

hand.
